# Quantum random number generator using a cloud superconducting quantum computer based on source-independent protocol

**DOI:** 10.1038/s41598-021-03286-9

**Published:** 2021-12-13

**Authors:** Yuanhao Li, Yangyang Fei, Weilong Wang, Xiangdong Meng, Hong Wang, Qianheng Duan, Zhi Ma

**Affiliations:** 1 State Key Laboratory of Mathematical Engineering and Advanced Computing, Zhengzhou, 450001 Henan China; 2 Henan Key Laboratory of Network Cryptography Technology, Zhengzhou, 450001 Henan China

**Keywords:** Quantum information, Quantum mechanics, Information theory and computation

## Abstract

Quantum random number generator (QRNG) relies on the intrinsic randomness of quantum mechanics to produce true random numbers which are important in information processing tasks. Due to the presence of the superposition state, a quantum computer can be used as a true random number generator. However, in practice, the implementation of the quantum computer is subject to various noise sources, which affects the randomness of the generated random numbers. To solve this problem, we propose a scheme based on the quantum computer which is motivated by the source-independent QRNG scheme in optics. By using a method to estimate the upper bound of the superposition state preparation error, the scheme can provide certified randomness in the presence of readout errors. To increase the generation rate of random bits, we also provide a parameter optimization method with a finite data size. In addition, we experimentally demonstrate our scheme on the cloud superconducting quantum computers of IBM.

## Introduction

Random number generators play an important role in many fields, such as cryptography^1^ and scientific simulations^2^. Different applications require different levels of randomness. For the applications which require the random numbers to be statistically unbiased, pseudo random number generators (PRNGs) or classical random number generators relying on deterministic algorithms or physical processes have been widely used^3,4^. Although their output sequences may appear random and usually have a perfect balance between 0 and 1, the predictability and strong long-range correlation may result in security loopholes when employed in some applications, particularly in cryptography and quantum key distribution^5,6^.

To solve this problem, based on the intrinsic uncertainty of quantum mechanics, quantum random number generators (QRNGs) can produce unpredictable random numbers and have attracted great attention in the past few years. Nowadays, many QRNG protocols implemented in optics have been proposed by using different randomness sources, including single photon detection^7–9^, vacuum state fluctuation^10–12^, laser phase fluctuation^13,14^ and amplified spontaneous emission noise^15,16^. There are already some commercial QRNG products implementing these protocols. In general, a QRNG system consists of two parts, a randomness source and a measurement unit. The randomness source emits the superposition state in the measurement basis whose measurement outcome is unpredictable to produce random numbers. For example, for the superposition state $|+\rangle =(|0\rangle +|1\rangle )/\sqrt{2}$, the results of projection measurements onto the $\{|0\rangle , |1\rangle \}$ basis of the state are purely random and ideally form the random numbers.

On the other hand, quantum computers based on different physical implementations have been rapidly developed^17^, including superconducting quantum circuits^18,19^, nuclear magnetic resonance^20,21^ and optical systems^22^. Some companies have launched cloud quantum computers which enable users to send quantum programs to use quantum computer^23,24^. Furthermore, due to the presence of superposition state, a quantum computer can be considered as an unbiased QRNG to generate random numbers^25^. Generally, a quantum bit (qubit) can be prepared in the superposition state $|+\rangle =(|0\rangle +|1\rangle )/\sqrt{2}$ by applying the Hadamard gate on the initial state $|0\rangle$. Thus, repeating measurements on the qubit in the $\{|0\rangle ,|1\rangle \}$ basis, the numbers 0 and 1 can be obtained with equal probabilities. Compared with the PRNG based on conventional digital computers, the QRNG based on quantum computers does not require random seeds, in which the risk of the predictability of output sequence can be avoided.

However, the imperfections of realistic devices and the presence of hardware noise may leave security loopholes that influence the randomness of generated random numbers in practice. To enhance the security of QRNG, self-testing or device-independent (DI) QRNGs are proposed which do not need to trust the generated quantum state and the measurement devices^26–28^. By observing the violation of the Bell inequality, the randomness of source can be guaranteed and true random numbers can be obtained. Unfortunately, realizing the practical implementation is difficult due to the requirement of a loophole-free Bell test, and the random number generation rate is very low, which cannot satisfy the demands of practical applications. To increase the generation rate and make the protocols more practical, a semi-self-testing or semi-device-independent (SDI) QRNG scheme with trusted part of the physical devices is proposed which presents a trade-off between the generation rate and the security of certified randomness^29–31^.

Among SDI-QRNG schemes, source-independent (SI) QRNG has gathered lots of attention^32–34^. With reasonable assumptions that measurement devices are well-calibrated and the source is untrusted, the SI-QRNG can generate secure random numbers and achieve considerable random number generation rates. Most of the SI-QRNG protocols are realized by the quantum optics devices. Utilizing a laser as a randomness source, Zhu *et al.* proposed a SI-QRNG scheme and realized a randomness generation rate of over 5000 bps^34^, and Marco *et al.* proposed a continuous-variable version of SI-QRNG protocol and realized a generation rate of 17 Gbps^35^.

In addition to the optics based SI-QRNG, the method of SI-QRNG protocol can also be performed on the quantum computer. The existing quantum computers are noisy and vulnerable to various types of errors, which causes errors in the preparation of superposition state $|+\rangle$ and affects the randomness of random numbers. For the conventional QRNG implementations, which output random numbers by directly measuring the quantum superposition state $|+\rangle$, the randomness of the output data cannot be well estimated due to the difficulty to model devices precisely. Although the random numbers generated by quantum computers can pass statistical testing after the combination of the von Neumann^36^ and Samuelson extractors^37^, the final random number extraction rate cannot be given and the randomness of the random numbers cannot be certified^38^.

SI-QRNG protocol does not require accurate characterization of the equipment to estimate the randomness of generated random numbers under the situation that the measurement error is known. Although the measurement error is timely varied, one’s own quantum computer can monitor the measurement error on time. The final extraction rate of random numbers can be given by estimating the error in the preparation of superposition state $|+\rangle$. Motivated by the original SI-QRNG protocol^34^, we propose a scheme that can guarantee the randomness of random numbers generated by noisy quantum computers. Rather than focusing on the influence of the source controlled by the adversary on the random numbers, we explore how many random bits can be extracted from raw data in the case of imperfect quantum gates. Using the cloud superconducting quantum computer of IBM, we experimentally examine the effectiveness of the proposed protocol based on the SI-QRNG protocol.

The rest of this article is organized as follows. In second section, the protocol based on SI-QRNG using cloud superconducting quantum computer is briefly introduced. In third section, we analyze the protocol, where the final extracted number of random bits is further given in the presence of readout errors, and the estimation method and optimization of the parameter are provided. In fourth section, an experimental demonstration of the scheme is performed with the cloud superconducting quantum computer of IBM. Finally, we conclude this paper in fifth section.

## QRNG based on source-independent protocol

In the quantum computer, the initial state of a qubit is generally prepared in $|0\rangle$ and can be represented with state vector as $$\begin{bmatrix} 1&0\end{bmatrix}^{T}$$ (T = Transpose). By applying $RY(\pi /2)$ gate, the superposition state $|+\rangle =(|0\rangle +|1\rangle )/\sqrt{2}$ can be acquired, in which $RY(\pi /2)$ gate performs $\pi /2$ rotation around Y-axis in the Bloch sphere and can be expressed with $$\frac{1}{\sqrt{2}}\begin{bmatrix} 1 &{} -1 \\ 1 &{} 1 \end{bmatrix}$$. If the quantum computer is noiseless and the quantum operations are perfect, the measurement results in the computational basis $|0\rangle$ and $|1\rangle$ should be uniformly random based on the mathematical axiom of quantum mechanics. However, the initial state, quantum gate and readout of each qubit may be impacted by hardware noise, which causes the temporal correlation between the output bits^39^. Thus, in the QRNG based on quantum computer, it is important to calculate how many random bits can be extracted from the raw data.

**Figure 1:**
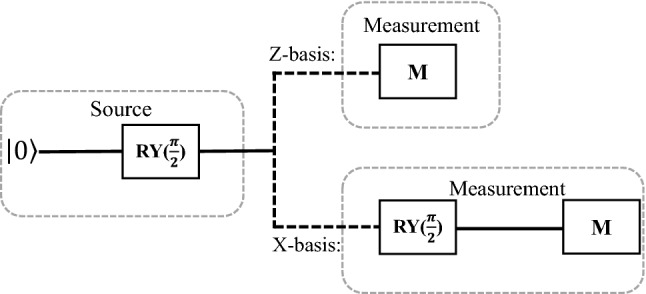
Quantum circuits for the QRNG based on SI protocol. The qubit state is randomly measured in the X-basis or Z-basis.

Motivated by the optics based SI-QRNG protocol proposed in Ref.^34^, we implement the protocol based on SI-QRNG using the quantum computer, as shown in Fig. 1. The detailed steps of the protocol are as follows:

1. Source: By applying $RY(\pi /2)$ gate on the initial state $|0\rangle$, a qubit is prepared in superposition state $|+\rangle =(|0\rangle +|1\rangle )/\sqrt{2}$.
2. Random sampling: By utilizing a short random seed, we randomly choose the X basis quantum circuit or Z-basis quantum circuit to measure the quantum superposition state $|+\rangle$. By adding a $RY(\pi /2)$ gate to the circuits, the Z-basis measurement can be converted into X-basis measurement, where $X=\{|0\rangle \pm |1\rangle /\sqrt{2}\}$ and $Z=\{|0\rangle ,|1\rangle \}$. In this process, the quantum circuit is executed *n* times, including $n_{x}$ times in the X-basis quantum circuit and $n_{z}$ times in the Z-basis quantum circuit, where $n=n_{x}+n_{z}$.
3. Parameter estimation: The quantum state emitted by the source should be $|+\rangle$ state when the quantum computer system is noiseless. The measurement result of $|+\rangle$ is $|1\rangle$ and the result of $|-\rangle$ is $|0\rangle$ in the X-basis, respectively. Therefore, a result of $|0\rangle$ means an error. We estimate the bit error rate $e_{bx}$ in the X-basis measurement and the statistical deviation is denoted by *o*, the preparation error of $|+\rangle$ state in the Z-basis $e_{z}$ can be estimated by
  1 $$\begin{aligned} e_{z}\le e_{bx}+o. \end{aligned}$$
  *o* is the deviation due to statistical fluctuations which is bounded by^34^
  2 $$\begin{aligned} \varepsilon _{e}=Prob(e_{z}>e_{bx}+o)\le \frac{1}{\sqrt{q_{x}(1-q_{x})e_{bx}(1-e_{bx})n}}2^{-n\zeta (o)}, \end{aligned}$$
  where $\zeta (o)=H(e_{bx}+o+q_{x}o)-q_{x}H(e_{bx})-(1-q_{x})H(e_{bx}+o)$, $q_{x}=n_{x}/n$ is the rate of X-basis choice and $H(x)=-xlog_{2}(x)-(1-x)log_{2}(1-x)$ represents the Shannon entropy function. If the value of $e_{bx}+o$ exceeds 0.5, we abort the protocol.
4. Randomness generation: The measurement results of the Z-basis are used to generate random numbers. We perform Z-basis quantum circuit $n_{z}$ times to generate $n_{z}$ random bits.
5. Randomness extraction: To extract true random numbers, the Topelitz-matrix hashing method is used. The number of final random bits is^34^
  3 $$\begin{aligned} K\ge n_{z}-n_{z}H(e_{z})-t_{e}, \end{aligned}$$
  where $2^{-t_{e}}$ is the failure probability of the randomness extraction^40^. In practice, to construct a Toeplitz matrix of size $n_{z} \times [n_{z}-n_{z}H(e_{z})-t_{e}]$ for randomness extraction, the length of $n_{z}+n_{z}-n_{z}H(e_{z})-t_{e}$ random bits is required. According to the leftover hash lemma^41^, the final output random bits are not affected by the random bits used in the construction of the Topelitz matrix.

Note that there is also another method to estimate the number of final random bits. Vallone *et al.*^42^ performed a complete finite key analysis of the protocol where the conditional min-entropy of the measurement results of Z-basis can be bounded by using the Rényi entropy of order 1/2 of the measurement results of X-basis $H_{1/2}(\{n_{x}\})$. Thus, by estimating the max-entropy $H_{1/2}(\{n_{x}\})$, the number of final random bits can be determined. In principle, both the methods in Ref.^34^ and^42^ can be used to estimate the number of final random bits. We choose the method in Ref.^34^ in our protocol.

## Analysis

In this section, we analyze the randomness of QRNG based on the quantum computer. Due to the presence of noise in the quantum computer, the readout, initial state and quantum gate are imperfect and thus impact the number of extracted random bits. The number of final random bits *K*, which is given by Eq. (3), does not consider the effect of readout errors. We first calculate the number of final random bits with different readout errors in “The number of extracted random bits in the presence of readout error” section. Then, to calculate the number of final random bits, a method for estimating the upper bound of $e_{z}$, i.e., the error in the preparation of quantum superposition state, is given out in “[Sec Sec5]” section. Finally, considering the influence of the finite data size on the parameter estimation, the ratio of X-basis measurements is optimized in “[Sec Sec6]” section.

### The number of extracted random bits in the presence of readout error

In practice, the readout operation of a quantum state is imperfect. Generally, the readout errors of $|0\rangle$ and $|1\rangle$ are different, which leads to the asymmetry in the 1/0 ratio of measurement outcomes. Thus, the influence of readout error on the number of extracted random numbers cannot be ignored. In the SI-QRNG based on optics, the influence of detector imperfection on the generated random numbers is analyzed in detail^43^. Motivated by this method, we analyze the scenario that the readout errors of $|0\rangle$ and $|1\rangle$ are different in the quantum computer and recalculate the number of final extracted random bits.

**Figure 2:**
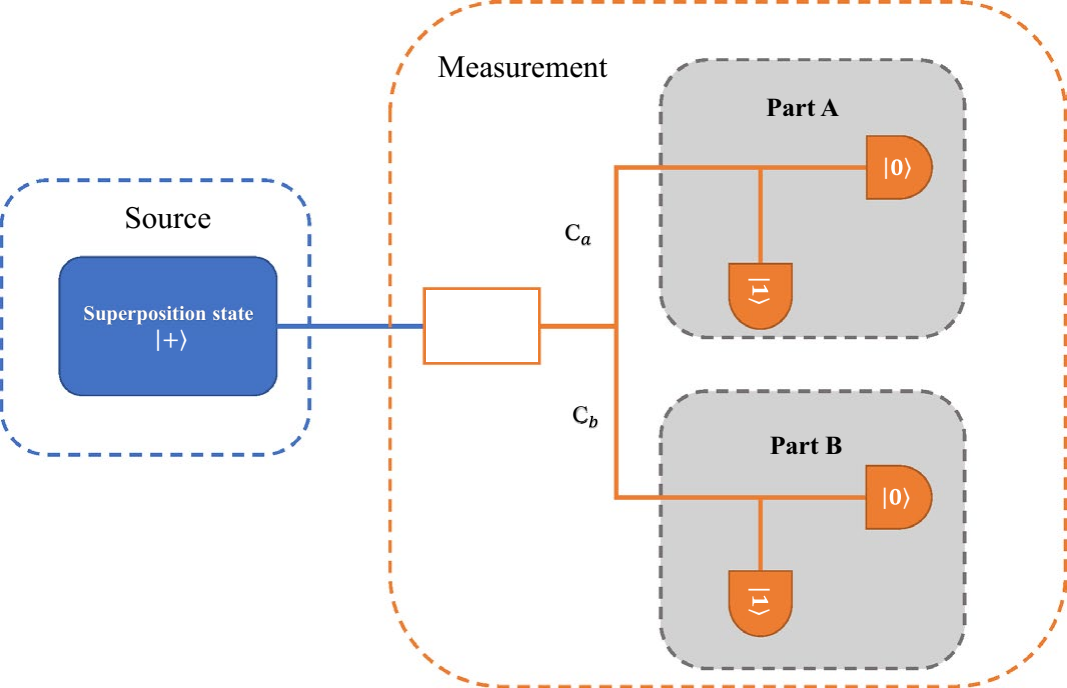
Equivalence of SI-QRNG under the different readout errors of a quantum state. The readout of a quantum state is equivalent to the superposition of two extreme parts: Part A and Part B. The readout errors of $|0\rangle$ and $|1\rangle$ are equal in Part A and are completely different in Part B, where the probabilities of occurrence of Part A and Part B are $c_{a}$ and $c_{b}$, respectively.

The readout error of $|0\rangle$, $r_{0}$, means the probability to output $|1\rangle$ while the actual state should be $|0\rangle$. Similarly, the readout error of $|1\rangle$, $r_{1}$, means the probability to output $|0\rangle$ while the actual state should be $|1\rangle$. When $r_{0}$ and $r_{1}$ are equal, the numbers of 1 and 0 in the measurement outcomes of superposition state $|+\rangle$ are equal and the randomness of the output bits cannot be affected by the readout error. Thus, the readout process of a qubit can be equivalent to the superposition of two parts, as shown in Fig. 2. One is the case that the readout errors of $|0\rangle$ and $|1\rangle$ are equal, and the probability of occurrence of Part A is $c_{a}$. The other one is the case that the readout errors of $|0\rangle$ and $|1\rangle$ are completely different and the probability of occurrence of Part B is $c_{b}$, in which the readout error of one quantum state is 1 and the readout error of another quantum state is 0. Therefore, no genuine randomness can be extracted from Part B. The relationship between $c_{a}$ and $c_{b}$ is $c_{a}+c_{b}=1$. Moreover, the readout error should fulfill

4 $$\begin{aligned} {\left\{ \begin{array}{ll} r_{0}=c_{a}r_{0,A}+c_{b}r_{0,B}\\ r_{1}=c_{a}r_{1,A}+c_{b}r_{1,B}, \end{array}\right. } \end{aligned}$$

where $r_{i,j}$ denotes the readout error of quantum state $|i\rangle$ for Part *j* with $i\in \{0,1\}$ and $j\in \{A,B\}$. Without loss of generality, we assume that the readout error $r_{0}$ is no larger than $r_{1}$. Due to the relationships $r_{0,A}=r_{1,A}$, $r_{0,B}=0$ and $r_{1,B}=1$, Eq. (4) can be simplified as

5 $$\begin{aligned} {\left\{ \begin{array}{ll} r_{0}=c_{a}r_{0,A}\\ r_{1}=c_{a}r_{1,A}+c_{b}. \end{array}\right. } \end{aligned}$$

Furthermore, we can obtain that $c_{b}=r_{1}-r_{0}$ and $c_{a}=1-c_{b}=1-(r_{1}-r_{0})$. Because random numbers cannot be extracted from the output bits in Part B, the mutual information between legitimate user (Q) and any third party user (E) is

6 $$\begin{aligned} I(Q:E)=c_{a}H(e_{z})+1-c_{a}=(1-r_{1}+r_{0})H(e_{z})+r_{1}-r_{0}. \end{aligned}$$

Therefore, we can rewrite the number of extracted random bits as

7 $$\begin{aligned} K_{final}=n_{z}[1-I(Q:E)]-t_{e}=(1-r_{1}+r_{0})[n_{z}-n_{z}H(e_{z})]-t_{e}. \end{aligned}$$

By observing Eqs. (3) and (7), we can find that the two formulas are the same when $r_{1}=r_{0}$ (i.e., the readout error is the same for $|0\rangle$ and $|1\rangle$).

### The estimation of upper bound of $e_{z}$

In practice, the preparation of superposition state $|+\rangle$ is affected by various noises, which results in errors and affects the random number generation rate. To determine how many random bits can be extracted from the raw data, we need to estimate the upper bound of parameter $e_{z}$, i.e., the errors in the preparation of $|+\rangle$ state. As shown in Fig. 1, $e_{z}$ can be mainly divided into two parts, one is the errors in the preparation of initial state $|0\rangle$, and the other is the errors in the operation of single-qubit $RY(\pi /2)$ gate. Through experimental measurements, the errors in the preparation of the initial state can be determined. And one’s own quantum computer can obtain the initialization error on time. According to Eq. (1), the upper bound of $e_{z}$ can be determined with the estimation of $e_{bx}$. Thus, to estimate the upper bound of $e_{z}$, the value of $e_{bx}$ should be firstly estimated. In this subsection, we consider two cases, one is to consider the errors in quantum gate and readout, and the other is to consider the errors in the initial state, quantum gate and readout.

In our protocol, only the single-qubit gate $RY(\pi /2)$ is used. Due to the presence of various noise and the imperfection of control mechanism, $RY(\pi /2)$ gate exits deviations of the rotation angle around the Y-axis and the axis of rotation. In this case, the actual $RY(\pi /2)$ gate can be equivalent to the superposition of $RY(\theta )$ gate and $RZ(\theta )$ gate. Denoting that the deviation of the rotation angle around the Y-axis is $\delta$ and the rotation angle around the Z-axis is $\phi$. Therefore, the actual rotation angle around the Y-axis is $\frac{\pi }{2}+\delta$ and the $RY(\frac{\pi }{2}+\delta )$ gate can be represented by square matrices, i.e., $$RY(\frac{\pi }{2}+\delta )=\begin{bmatrix} cos(\frac{\pi }{4}+\frac{\delta }{2}) &{} -sin(\frac{\pi }{4}+\frac{\delta }{2}) \\ sin(\frac{\pi }{4}+\frac{\delta }{2}) &{} cos(\frac{\pi }{4}+\frac{\delta }{2}) \end{bmatrix}$$. The $RZ(\phi )$ gate is expressed with $$\begin{bmatrix} e^{-i\frac{\phi }{2}}&{} 0 \\ 0 &{} e^{i\frac{\phi }{2}} \end{bmatrix}$$.

Case 1: The errors in quantum gate and readout are considered.

To prepare the superposition state $|+\rangle$, the first $RY(\pi /2)$ gate is performed on the initial state $|0\rangle$ , which is equivalent to the superposition of $RY(\frac{\pi }{2}+\delta )$ gate and $RZ(\phi )$ gate, resulting in the quantum state

8 $$\begin{aligned} |\varphi _{1}\rangle =\frac{cos\left( \frac{\pi }{4}+\frac{\delta }{2}\right) e^{-i\frac{\phi }{2}}+sin\left( \frac{\pi }{4}+\frac{\delta }{2}\right) e^{i\frac{\phi }{2}}}{\sqrt{2}}|+\rangle + \frac{cos\left( \frac{\pi }{4}+\frac{\delta }{2}\right) e^{-i\frac{\phi }{2}}-sin\left( \frac{\pi }{4}+\frac{\delta }{2}\right) e^{i\frac{\phi }{2}}}{\sqrt{2}}|-\rangle = \begin{bmatrix} cos\left( \frac{\pi }{4}+\frac{\delta }{2}\right) e^{-i\frac{\phi }{2}}&sin\left( \frac{\pi }{4}+\frac{\delta }{2}\right) e^{i\frac{\phi }{2}}\end{bmatrix}^{T}. \end{aligned}$$

Therefore, the prepared quantum state is in the superposition of $|+\rangle$ state and $|-\rangle$ state, where the probability of $|+\rangle$ state is $\frac{1+cos\delta cos\phi }{2}$ and the probability of $|-\rangle$ state is $\frac{1-cos\delta cos\phi }{2}$, respectively. Applying the same $RY(\frac{\pi }{2}+\delta )$ gate and $RZ(\phi )$ gate on the quantum state $|\varphi _{1}\rangle$, the resulting quantum state is

9 $$\begin{aligned} |\varphi _{2}\rangle =\left( \left( \frac{1-sin(\delta )}{2}\right) (cos\phi -isin\phi )-\left( \frac{1+sin(\delta )}{2}\right) \right) |0\rangle +\left( \left( \frac{cos(\delta )}{2}\right) (cos\phi -isin\phi +1)\right) |1\rangle . \end{aligned}$$

Finally, we can obtain $|0\rangle$ or $|1\rangle$ to measure the quantum state $|\varphi _{2}\rangle$, where the probability of $|0\rangle$ is $(\frac{sin^{2}\delta +1-cos\phi +sin^{2}\delta cos\phi }{2})$ and the probability of $|1\rangle$ is $\frac{cos^{2}\delta }{2}(1+cos\phi )$ in theory.

Furthermore, considering the readout error in the quantum computer, we can obtain

10 $$\begin{aligned} {\left\{ \begin{array}{ll} N_{0}=n_{0}(1-r_{0})+n_{1}r_{1}\\ N_{1}=n_{1}(1-r_{1})+n_{0}r_{0}, \end{array}\right. } \end{aligned}$$

where $N_{0}$ and $N_{1}$ are the numbers of 0 and 1 in the results of X-basis measurement with readout error which satisfies $N_{0}+N_{1}=n_{x}$, $n_{0}$ and $n_{1}$ represent the numbers of 0 and 1 in the results of X-basis measurement without readout error, respectively. $n_{0}$ and $n_{1}$ can be expressed as

11 $$\begin{aligned} {\left\{ \begin{array}{ll} n_{0}=\left( \frac{sin^{2}\delta +1-cos\phi +sin^{2}\delta cos\phi }{2}\right) n_{x}\\ n_{1}=\frac{cos^{2}\delta }{2}(1+cos\phi )n_{x}. \end{array}\right. } \end{aligned}$$

In our protocol, the result of $|-\rangle$ state in the randomness source is defined as the preparation error of superposition state, so the error $e_{bx}$ is equal to the probability of $|-\rangle$ state, i.e., $e_{bx}=\frac{1-cos\delta cos\phi }{2}$.

By solving Eq. (10), the value of $n_{0}$ and $n_{1}$ can be obtained. In a real quantum computer, the deviation of rotation angle around the Y-axis $\delta$ and around the Z-axis $\phi$ are both in $(-\frac{\pi }{2},\frac{\pi }{2})$, so $0<{cos\phi }<1$. According to Eq. (11), we can obtain $cos^{2}\delta =\frac{2n_{1}}{n_{x}(1+cos\phi )}$. Based on the expression for $cos^{2}\delta$ and the range value of $cos\phi$, the range of $\delta$ can be determined which satisfies $\frac{n_{1}}{n_{x}}<cos^{2}\delta <\frac{2n_{1}}{n_{x}}$. Given a value of $\delta$, the value of $\phi$ can also be determined with Eq. (11). Therefore, the preparation error of $|+\rangle$ in the X-basis measurement can be calculated with $e_{bx}=\frac{1-cos\delta cos\phi }{2}$.

Case 2: The errors in the initial state, quantum gate and readout are considered.

Due to the presence of errors in preparation of initial state, the initial state can be represented with $|\varphi _{0}\rangle =\alpha |0\rangle +\beta |1\rangle$, where $|\alpha |^{2}+|\beta |^{2}=1$, and the value of $\alpha$ and $\beta$ can be obtained by measuring the initial state directly. In the computational basis, the state can be written as vector $$\begin{bmatrix} \alpha&\beta \end{bmatrix}^{T}$$. The first $RY(\pi /2)$ gate is applied on the initial state $|\varphi _{0}\rangle$ to prepare the state $|+\rangle$, resulting in quantum state

12 $$\begin{aligned} \begin{aligned} |\varphi _{1}\rangle&=\frac{e^{-i\frac{\phi }{2}}\left( \alpha cos\left( \frac{\pi }{4}+\frac{\delta }{2}\right) -\beta sin\left( \frac{\pi }{4}+\frac{\delta }{2}\right) \right) + e^{i\frac{\phi }{2}}\left( \alpha sin\left( \frac{\pi }{4}+\frac{\delta }{2}\right) +\beta cos\left( \frac{\pi }{4}+\frac{\delta }{2}\right) \right) }{\sqrt{2}}|+\rangle \\&\quad +\frac{e^{-i\frac{\phi }{2}}\left( \alpha cos\left( \frac{\pi }{4}+\frac{\delta }{2}\right) -\beta sin\left( \frac{\pi }{4}+\frac{\delta }{2}\right) \right) -e^{i\frac{\phi }{2}}\left( \alpha sin\left( \frac{\pi }{4}+\frac{\delta }{2}\right) +\beta cos\left( \frac{\pi }{4}+\frac{\delta }{2}\right) \right) }{\sqrt{2}}|-\rangle \\&=\begin{bmatrix}e^{-i\frac{\phi }{2}}\left( \alpha cos\left( \frac{\pi }{4}+\frac{\delta }{2}\right) -\beta sin\left( \frac{\pi }{4}+\frac{\delta }{2}\right) \right)&e^{i\frac{\phi }{2}}\left( \alpha sin\left( \frac{\pi }{4}+\frac{\delta }{2}\right) +\beta cos\left( \frac{\pi }{4}+\frac{\delta }{2}\right) \right) \end{bmatrix}^{T}. \end{aligned} \end{aligned}$$

Thus, the probability of $|+\rangle$ state and $|-\rangle$ state are $cos\phi (\alpha ^{2}cos\delta -\beta ^{2}cos\delta -\alpha \beta sin\delta )$ and $1-cos\phi (\alpha ^{2}cos\delta -\beta ^{2}cos\delta -\alpha \beta sin\delta )$, respectively. To measure the quantum state on the X-basis, the same $RY(\frac{\pi }{2}+\delta )$ gate and $RZ(\phi )$ gate are performed on the quantum state $|\varphi _{1}\rangle$, and the quantum state $|\varphi _{2}\rangle$ is obtained with

13 $$\begin{aligned} \begin{aligned} |\varphi _{2}\rangle&=\left[ e^{-i\phi } cos\left( \frac{\pi }{4}+\frac{\delta }{2}\right) \left( \alpha cos\left( \frac{\pi }{4}+\frac{\delta }{2}\right) -\beta sin\left( \frac{\pi }{4}+\frac{\delta }{2}\right) \right) -sin\left( \frac{\pi }{4}+\frac{\delta }{2}\right) \left( \alpha sin\left( \frac{\pi }{4}+\frac{\delta }{2}\right) +\beta cos\left( \frac{\pi }{4}+\frac{\delta }{2}\right) \right) \right] |0\rangle \\&\quad +\left[ e^{i\phi }cos\left( \frac{\pi }{4}+\frac{\delta }{2}\right) \left( \alpha sin\left( \frac{\pi }{4}+\frac{\delta }{2}\right) +\beta cos\left( \frac{\pi }{4}+\frac{\delta }{2}\right) \right) +sin(\frac{\pi }{4}+\frac{\delta }{2})\left( \alpha cos\left( \frac{\pi }{4}+\frac{\delta }{2}\right) +\beta sin\left( \frac{\pi }{4}+\frac{\delta }{2}\right) \right) \right] |1\rangle \end{aligned} \end{aligned}$$

By measuring the quantum state $|\varphi _{2}\rangle$, we can obtain that the probability of $|0\rangle$ state is $\alpha ^{2}+\frac{1}{2}(\beta ^{2}-\alpha ^{2})(cos^{2}\delta +cos \phi cos^{2}\delta )+\frac{1}{2}\alpha \beta sin2\delta (cos\phi +1)$ and the probability of $|1\rangle$ state is $\beta ^{2}+\frac{1}{2}(\alpha ^{2}-\beta ^{2})(cos^{2}\delta +cos \phi cos^{2}\delta )-\frac{1}{2}\alpha \beta sin2\delta (cos\phi +1)$ in theory. The error $e_{bx}$ is equal to the probability of $|-\rangle$ state, i.e., $e_{bx}=1-cos\phi (\alpha ^{2}cos\delta -\beta ^{2}cos\delta -\alpha \beta sin\delta )$.

Based on Eq. (10), the numbers of 0 and 1 in the results of X-basis measurement without readout errors, i.e., $n_{0}$ and $n_{1}$, can be determined. According to the probabilities of 0 and 1 by measuring the quantum state $|\varphi _{2}\rangle$, $n_{0}$ and $n_{1}$ can be expressed as

14 $$\begin{aligned} {\left\{ \begin{array}{ll} n_{0}=(\alpha ^{2}+\frac{1}{2}(\beta ^{2}-\alpha ^{2})(cos^{2}\delta +cos \phi cos^{2}\delta )+\frac{1}{2}\alpha \beta sin2\delta (cos\phi +1))n_{x}\\ n_{1}=(\beta ^{2}+\frac{1}{2}(\alpha ^{2}-\beta ^{2})(cos^{2}\delta +cos \phi cos^{2}\delta )-\frac{1}{2}\alpha \beta sin2\delta (cos\phi +1))n_{x} \end{array}\right. }. \end{aligned}$$

Similar with the analysis of Case 1, the deviations of rotation angle around the Y-axis $\delta$ and around the Z-axis $\phi$ are both in $(-\frac{\pi }{2},\frac{\pi }{2})$ and $0<{cos\phi }<1$. With Eq. (14) and the range value of $cos\phi$, the range of $\delta$ can be determined which satisfies $\frac{\alpha ^{2}-\frac{n_{0}}{n_{x}}}{2}<\frac{1}{2}(\alpha ^{2}-\beta ^{2})cos^{2}\delta -\frac{1}{2}\alpha \beta sin2\delta <\alpha ^{2}-\frac{n_{0}}{n_{x}}$. Given a value of $\delta$, the value of $\phi$ can also be determined with Eq. (14). Therefore, the preparation error of $|+\rangle$ in the X-basis measurement can be calculated with $e_{bx}=1-cos\phi (\alpha ^{2}cos\delta -\beta ^{2}cos\delta -\alpha \beta sin\delta )$.

**Figure 3:**
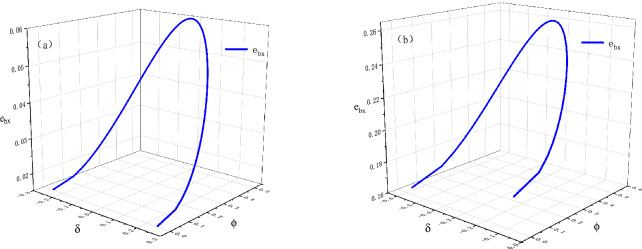
Relationships between $e_{bx}$, $\delta$ and $\phi$. Simulated results with varying $\delta$ and $\phi$. (**a**) Case 1; (**b**) Case 2.

**Figure 4:**
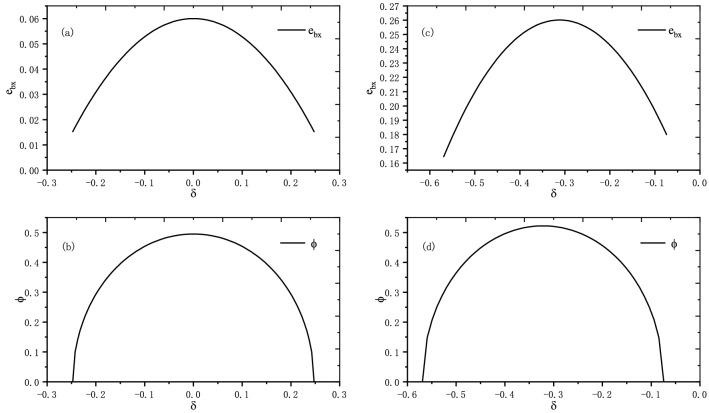
(**a**, **c**) Projection of the *xz* plane of Fig. 3a and b. Relationship between rotation angle error $\delta$ and preparation error of $|+\rangle$ state $e_{bx}$. (**b**, **d**) Projection of the *xy* plane of Fig. 3a and b. Relationship between errors in the rotation angle around Y-axis $\delta$ and Z-axis $\phi$.

For example, suppose $\frac{N_{0}}{n_{x}}=0.151$, $\frac{N_{1}}{n_{x}}=0.849$, $r_{0}=0.05$, $r_{1}=0.1$, $\alpha ^{2}=0.9$ and $\beta ^{2}=0.1$, we can obtain $\frac{n_{0}}{n_{x}}=0.06$ and $\frac{n_{1}}{n_{x}}=0.94$ with Eq. (10). Based on the expression of $\delta$, the range value of $\delta$ can be calculated and the corresponding results are $(-0.24747,0.2474)$ and $(-0.56921,-0.07428)$ in Case 1 and Case 2, respectively. With (11) and Eq. (14), the deviation of rotation angle around the Z-axis $\phi$ is determined in the two Cases. Moreover, the relationship between $\delta$ and $\phi$ is shown in Fig. 4b and d. Utilizing the determined value of $\delta$ and $\phi$, the error $e_{bx}$ can be calculated in Case 1 and Case 2, and the relationship between $\delta$, $\phi$ and $e_{bx}$ is shown in Fig. 3. Figure 4a and c shows the relationship between $\delta$ and $e_{bx}$ in Case 1 and Case 2, respectively. In Case 1, we can discover that the parameter $e_{bx}$ has a maximum value when $\delta =0$, i.e., $e_{bx}\le 0.05998$. In Case 2, the parameter $e_{bx}$ also has a maximum value, i.e., $e_{bx}\le 0.26016$. The value of $e_{bx}$ calculated in Case 2 is larger than that in Case 1, which means that the errors in preparation of initial state have effects on $e_{bx}$. Then, according to Eq. (1), the bound of error $e_{z}$ can be determined.

### Optimization of parameter $q_{x}$

In the cloud superconducting quantum computer of IBM, the quantum circuit is repeatedly sent to the real devices. The running time directly affects the final data and parameter estimation. To increase the final generation rate of the QRNG and improve the security of the QRNG protocol, parameters should be optimized. Based on the parameter optimization method in Ref.^34^, we consider the influence of the finite data size on the parameter estimation and optimize the ratio of X-basis measurements $q_{x}$.

In our protocol, the measurements results of superposition state $|+\rangle$ in the Z basis are used to generate random numbers, and the errors in the preparation of superposition state $|+\rangle$ can be estimated by the measurement results of X-basis. Based on the method introduced in “[Sec Sec6]” section, $e_{z}$ can be well approximated by $e_{bx}$ with an infinite data size. However, due to the statistical fluctuations, the parameter $e_{z}$ cannot be estimated accurately and the method of approximating is crucial. The parameter $e_{z}$ is estimated by Eq. (1) and the statistical fluctuation *o* is bounded by Eq. (2). According to Eq. (2), there is a trade-off between $q_{x}$ and *o* for the ratio of the final random bit length over the raw data size given that $\varepsilon _{e}$ is fixed. Generally, the failure probability $\varepsilon _{e}$ is picked to be a small value. Hence, the value of $q_{x}$ should be optimized for the randomness extraction rate and follows the condition:

15 $$\begin{aligned} \begin{aligned}{}&\mathbf{Max}: K_{final},\\&\mathbf{s}.t.: \varepsilon _{e}=Prob(e_{z}>e_{bx}+o)\le \frac{1}{\sqrt{q_{x}(1-q_{x})e_{bx}(1-e_{bx}n)}}2^{-n\zeta (o)} \end{aligned} \end{aligned}$$

**Figure 5:**
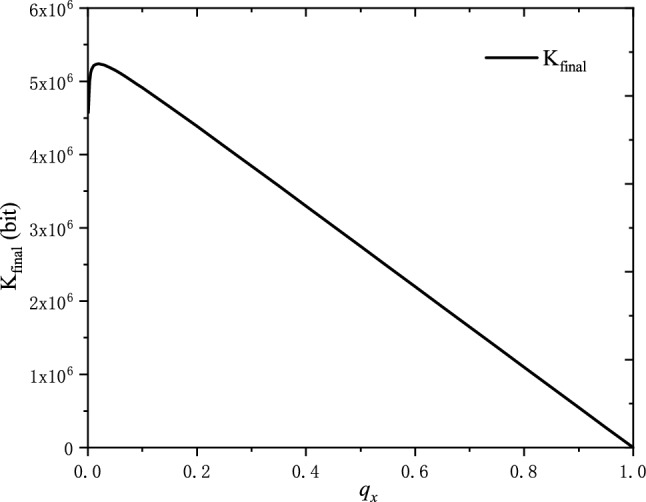
Relationship between basis choice rate $q_{x}$ and final extracted random bits $K_{final}$. Here, we set $e_{bx}=0.05$, $r_{0}=0.05$, $r_{1}=0.1$ and $n=8.192\times 10^{6}$.

With the method of the numerical solution, the optimized $q_{x}$ can be obtained. In the cloud superconducting quantum computer of IBM, the maximum executing number of a quantum circuit is 8192 times. Repeating the quantum circuit, the final number of executions *n* can be up to $8.192\times 10^{6}$ times. The value of $\varepsilon _{e}$ is $2^{-100}$ in our later data processing. Supposing that the preparation error of superposition sate in the X-basis $e_{bx}$ is 0.05, the readout error of $|0\rangle$ is $r_{0}=0.05$ and the readout error of $|1\rangle$ is $r_{0}=0.1$, we can compute the optimal $q_{x}$ for the final extracted random bits $K_{final}$, as shown in Fig. 5. The value of $K_{final}$ has a maximum value which means that the generation rate of random numbers can achieve a maximum value for a given condition.

## Experiment on the cloud superconducting quantum computer of IBM

In this section, we perform our proposed protocol on the cloud superconducting quantum computers of IBM to show its practicality. Since the error in the preparation of the initial sates is almost zero in the quantum computers of IBM, the error $e_{bx}$ can be estimated with the method in Case 1. In the quantum computer system, 8192 is the maximum number of uninterrupted shots available. For demonstration purposes, the basis choice is achieved by running the Z-basis measurement of the quantum circuit with 8192 times and the X-basis measurement of the quantum circuit with 251 times.

**Figure 6:**
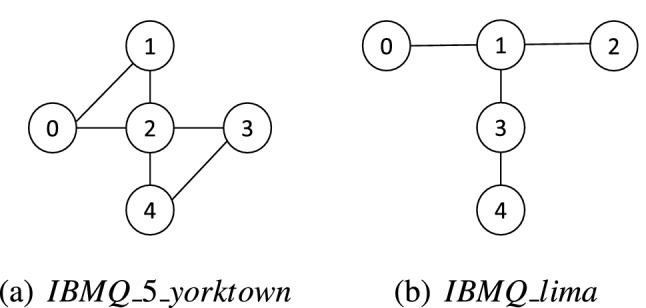
Device topology of $IBMQ\_5\_yorktown$ and $IBMQ\_lima$.

$IBMQ\_5\_yorktown$ and $IBMQ\_lima$ are used in the experiment where the device topologies are shown in Fig. 6^44^. $IBMQ\_5\_yorktown$ and $IBMQ\_lima$ both have five qubits and the readout error for each qubit is provided by Qiskit^45^. Without loss of generality, we select the qubit 0 ($Q_{0}$) of the two devices to execute the quantum circuit and the readout errors of $Q_{0}$ for these two devices are shown in Table 1.

**Table 1:** The readout errors of $Q_{0}$ for $IBMQ\_5\_yorktown$ and $IBMQ\_lima$.

Device	Readout error	
	$r_{0}$	$r_{1}$
$IBMQ\_5\_yorktown$	0.072	0.0394
$IBMQ\_lima$	0.0964	0.0122

By running the quantum circuits of SI-QRNG repeatedly, we obtain two sequences under each quantum computer device which are the results of the Z-basis measurement and X-basis measurement. The sequence $L_{z}$ of Z-basis measurement is used to extract random bits and its length $n_{z}$ is 819200. The other sequence $L_{x}$ is used to estimate the preparation error of superposition state $|+\rangle$ in the Z-basis $e_{bx}$ and its length $n_{x}$ is 25100.

In the sequence $L_{x}$ of $IBMQ\_5\_yorktown$, the number of 0 is $N_{0}=2669$ and the number of 1 is $N_{1}=22431$. According to Eq. (10) and the readout error of $Q_{0}$, we can obtain $n_{0}=299.8557$ and $n_{1}=24800.1443$. With Eq. (11), the value of $\delta$ is calculated which is between $-0.1095186$ and 0.1095186. Exploiting the expression for $e_{bx}$, we can obtain the maximum value of $e_{bx}$ is 0.011943. Based on Eqs. (1) and (2), the bound of $e_{z}$ is determined and equals to 0.0122442. Thus, the number of random bits $K_{yorktown}$ that can be extracted from the Z-basis measurement is 741006 which is calculated by using Eq. (7), and the corresponding random bits’ generation rate $r_{yorktown}$ is 0.9045. Utilizing the same method, the parameter $e_{z}$ and the final extracted random bits $K_{lima}$ in the $IBMQ\_lima$ device can be calculated. The numbers of 0 and 1 in the X-basis of $IBMQ\_lima$ are $N_{0}=1186$ and $N_{1}=23914$. By calculating, we can obtain $e_{bx}=0.039318$, $e_{z}=0.0397301$, $K_{lima}=621729$ and the corresponding random bits’ generation rate $r_{lima}$ is 0.7589.

After obtaining the raw data and the estimated $e_{z}$, we apply the Toeplitz matrix hashing on the raw data to obtain the final random numbers^46^. To evaluate the randomness of the final data, we perform the NIST Statistical Test on the final random numbers^47^. Since the length of the final data cannot satisfy some test items of the NIST Statistical Test, the final data is only subjected to the nine test items from the NIST Statistical Test which are the frequency test, frequency within a block test, runs test, longest runs within a block test, FFT test, approximate entropy test, Matrix Rank Test and the cumulative sums test (forward, backward). Each test item produces a corresponding P-value and the significance level $\alpha$ is set as 0.01 in our test. If the P-value $\ge$ $\alpha$, the final data is considered as true random numbers with $1-\alpha$ of confidence level. The results of the NIST Statistical Test on the two final sequences with a length of 600,000 bits are shown in Table 2. From Table 2, one can see that all the P-values are larger than 0.01, which indicates the final data of $IBMQ\_5\_yorktown$ and $IBMQ\_lima$ pass all test items.

**Table 2:** The NIST Statistical Test results and corresponding P-value of final data.

Test	$IBMQ\_5\_yorktown$	$IBMQ\_lima$
Frequence	0.706196	0.434013
Block frequency	0.444177	0.754105
Runs	0.760474	0.120287
Longest run	0.668568	0.653644
FFT	0.981097	0.915088
Approximate entropy	0.325238	0.162457
Rank	0.891846	0.653728
Cumulative sums (forward)	0.916711	0.782753
Cumulative sums (backward)	0.834860	0.628746
Result	Success	Success

**Figure 7:**
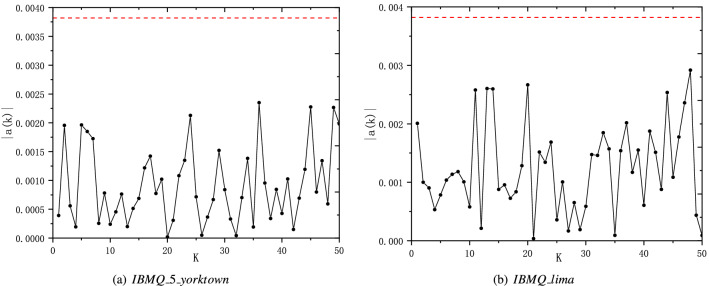
The absolute value of the autocorrelation function of the final data generated by $IBMQ\_5\_yorktown$ and $IBMQ\_lima$. The red dashed line is the three-standard-deviation line.

Furthermore, we calculate the autocorrelation coefficients of the final data to test the independence between neighboring bits of final data. The autocorrelation coefficient is defined as $a(k)=\frac{\Xi [(X_{i}-\mu )(X_{i+k}-\mu )]}{\sigma ^{2}}$, where $\Xi$ stands for expectation, $X_{i}$ denotes the value of the $i_{th}$ bit in the sequence, $\mu$ and $\sigma ^{2}$ are the average and the variance of the sequence^16^. The final data with length of $n_{l}$ is considered as true random numbers when all autocorrelation coefficients are greater than the three-standard-deviation value $a_{3\sigma }$ with $a_{3\sigma }=3/\sqrt{n_{l}}$. We choose a sequence with a length of 600,000 bits to perform the autocorrelation test and the corresponding $a_{3\sigma }$ is approximately 0.003873. The results of the autocorrelation test of the two final sequences are shown in Fig. 7. The red dashed line stands for the corresponding three-standard-deviation value $a_{3\sigma }$. It can be seen that all the absolute values of autocorrelation coefficients are below $a_{3\sigma }$. From the results of the NIST Statistical Test and autocorrelation test, we can know the randomness of the final data generated by $IBMQ\_5\_yorktown$ and $IBMQ\_lima$ can be guaranteed.

## Conclusion

Due to the presence of noise and the imperfection of the control mechanism, there exit errors in the initialization, quantum gate and readout in the quantum computer, which leads to the bias of the output data. Motivated by the SI-QRNG based on optics^34^, we propose and implement a QRNG scheme using a cloud superconducting quantum computer. Our proposed protocol can estimate the preparation error of superposition state $|+\rangle$ and give the final number of extracted random bits, which guarantees the security of generated random numbers. The readout errors of $|0\rangle$ and $|1\rangle$ are generally different in the quantum computer, which impacts the randomness of generated random numbers. Utilizing the method for solving the imperfection of detector in origin SI-QRNG protocol, we firstly give the final extracted number of random bits $K_{final}$ with readout error. Then, by further considering the errors in the preparation of initial state and quantum gate, the estimation methods for parameter $e_{z}$ are given, where the quantum gate error includes the deviation of the rotation angle around the Y-axis $\delta$ and the rotation angle around the Z-axis $\phi$. Moreover, we optimize the ratio of X-basis measurements $q_{x}$ to increase the final random number generation rate.

To prove the practicability of our protocol, we perform our proposed protocol on the cloud superconducting quantum computer of IBM. The random numbers generated by $IBMQ\_5\_yorktown$ and $IBMQ\_lima$ are post-processed by Toeplitz matrix hashing to obtain the final random numbers. The results of the NIST Statistical Test and autocorrelation test show that the final random numbers could be considered as true random numbers. Utilizing the SI-QRNG scheme, the error in the preparation of quantum superposition state $|+\rangle$ can be monitored, and we realize the generation of true random numbers in quantum computers with noise.
